# Identification of the Role of *NAT10* in the Regulation of Porcine Circovirus Type 2 Infection

**DOI:** 10.3390/vetsci12121160

**Published:** 2025-12-04

**Authors:** Ying Liu, Gang Zhou, Guolian Wang, Zhengchang Wu

**Affiliations:** 1 School of Life Science, Huaiyin Normal University, Huai'an 223001, China; 2 Huaiyin Institute of Agricultural Sciences in Xuhuai Regio, Huai'an 223000, China; 3 College of Animal Science and Technology, Yangzhou University, Yangzhou 225009, China

**Keywords:** porcine circovirus type 2, NAT10, transcriptome, virus replication

## Abstract

Porcine circovirus type 2 (PCV2) represents a principal infectious agent causing considerable economic detriment to swine production. N-acetyltransferase 10 (NAT10), which catalyzes N^4^-acetylcytidine (ac^4^C) deposition, has been implicated in regulating immune responses, RNA stability, and viral replication. However, its role in PCV2 infection remains unclear. In this study, we established a PCV2-infected PK15 cell model and observed a marked downregulation of NAT10 expression following infection. Functional assays demonstrated that NAT10 knockdown significantly suppressed PCV2 replication in PK15 cells. Comparative transcriptomic analysis revealed that NAT10 silencing altered the expression of 81 genes, predominantly involved in immune-related signaling pathways. Notably, integrative omics analysis identified NR1H4 as a potential downstream target of NAT10. Collectively, these findings elucidate the regulatory mechanism of NAT10 in PCV2 replication and provide new insights for identifying NAT10 as a potential biomarker and therapeutic target for PCV2 infection in pigs.

## Simple Summary

Porcine circovirus type 2 (PCV2) represents the foremost pathogenic factor underlying postweaning multisystemic wasting syndrome, which has resulted in marked economic ramifications for the pork production sector. However, the role of RNA ac^4^C modification in PCV2 replication remains largely unexplored. Here, the present work provides the initial characterization of NAT10’s functional significance and molecular underpinnings in governing PCV2 replication in PK15 cells. Our findings demonstrate that NAT10 exerts critical influence on PCV2 infectivity and illuminate novel perspectives regarding its therapeutic potential as an antiviral molecular target.

## 1. Introduction

Over 170 ribonucleotide modifications have been identified to date, and new types continue to emerge. RNA chemical modifications serve crucial regulatory purposes in governing RNA metabolism and downstream functions, influencing processes such as RNA splicing, localization, transport, translation, and stability [1]. Methylation modifications occupy a central position in this regulatory network, with N6-methyladenosine (m^6^A) and 5-methylcytosine (m^5^C) being particularly well-established as modulators of both host-encoded and virus-derived RNA activities [2,3,4]. In contrast, N4-acetylcytidine (ac^4^C), the only known acetylation modification on eukaryotic mRNAs, remains relatively underexplored. N-acetyltransferase 10 (NAT10), categorized as a GCN5-related N-acetyltransferase family member, has been recognized as the acetyltransferase governing catalytic processes for histone acetylation alongside ac^4^C RNA chemical modification. In humans and yeast, ac^4^C is crucial for tRNA translation and stability, as well as for rRNA biogenesis [5,6,7]. Recent advances using antibody-based RNA immunoprecipitation followed by deep sequencing (RIP-seq) have revealed the presence of ac^4^C in mRNA, suggesting that this modification enhances mRNA stability and translation efficiency [8]. Notably, previous studies have shown that NAT10 promotes the replication of several viruses, including HIV-1, influenza virus, Sindbis virus (SINV), enterovirus 71 (EV71), and Kaposi’s sarcoma-associated herpesvirus (KSHV), although the precise mechanisms remain unclear [9,10,11,12,13]. Given that NAT10 has also been implicated in type I interferon (IFN) signaling in a sepsis mouse model, it is plausible that this enzyme plays a broad regulatory role in antiviral innate immunity [14,15]. Emerging evidence further suggests that NAT10 may act as a negative regulator of both innate and adaptive immune responses. For instance, systemic lupus erythematosus (SLE) patients display reduced NAT10 levels in their CD4+ T cell populations when benchmarked against healthy subjects. In contrast, elevated NAT10 expression has been documented across various tumor entities, pointing toward its plausible participation in neoplastic immune evasion strategies [16,17,18,19,20]. Collectively, these findings suggest that NAT10 may influence the outcome of viral infections through its ability to modulate immune responses under various pathological conditions.

Porcine circoviruses (PCVs) represent small-sized, non-enveloped viral agents possessing circular single-stranded DNA genomes measuring 1.7–2.0 kb in length [21,22]. Molecular characterization has revealed four PCV genotypes circulating in pig populations [23]. Among these genotypes, PCV1 demonstrates avirulent characteristics, whereas PCV2 serves as the predominant pathogenic driver of porcine circovirus-associated diseases (PCVAD) [24]. Novel genotypes PCV3 and PCV4 have been isolated and characterized in recent epidemiological studies [25]. The porcine circovirus type 2 (PCV2) genome is approximately 1.7 kb in length and encodes 11 putative open reading frames (ORFs), six of which have been functionally characterized. Among these, ORF1 and ORF2 are considered the principal ORFs, encoding the replication-associated protein (Rep) and the capsid protein (Cap), respectively [26]. PCV2 infection causes immunosuppression in the host, predisposing pigs to secondary infections by multiple pathogens [27]. PCV2 DNA exhibits extensive environmental distribution and has been identified across multiple non-porcine animal species, encompassing rats, young cattle, minks, and foxes [28]. Therefore, understanding the mechanisms underlying PCV2 pathogenesis in host cells is of great importance. Recent studies on the epigenetic regulation of PCV2 infection have mainly focused on DNA methylation [29,30], histone acetylation [31], and m6A modification [32]. However, the involvement of NAT10-mediated N4-acetylcytidine (ac^4^C) modification in PCV2 replication has not been reported. In this study, we established a porcine kidney (PK15) cell model of PCV2 infection and observed a significant downregulation of the ac^4^C-related gene NAT10. Moreover, NAT10 knockdown was found to suppress PCV2 replication in PK15 cells. Comparative transcriptome analysis between the NAT10 knockdown and control groups revealed that NAT10 may regulate PCV2 replication by targeting NR1H4. Our findings highlight the role of ac^4^C modification in PCV2 replication and elucidate the molecular mechanism by which NAT10 participates in viral replication, providing new insights into the pathogenesis of PCV2 infection.

## 2. Materials and Methods

### 2.1. Cell Culture and PCV2 Viral Infection

The PK15 cell line (ATCC, CCL-33) was propagated in DMEM (Thermo, Waltham, MA, USA) enriched with 10% FBS (Bio-Channel, Nanjing, China) and 100 U/mL penicillin–streptomycin solution (Solarbio, Beijing, China). Cultures were maintained in a 37 °C incubator with a humidified 5% CO_2_ atmosphere. For infection experiments, a PCV2d strain archived in our laboratory was applied to PK15 cells at an MOI value of 1.

### 2.2. RNA Isolation, cDNA Synthesis and Quantitative PCR

Total cellular RNA was isolated utilizing TRIzol reagent (Thermo, Waltham, MA, USA). First-strand cDNA was generated using HiScript II Q Select RT SuperMix with gDNA Remover or miRNA 1st Strand cDNA Synthesis Kit (Vazyme, Nanjing, China) according to standard procedures. For genomic DNA preparation, the FastPure Cell/Tissue DNA Isolation Mini Kit (Vazyme, Nanjing, China) was utilized as per manufacturer’s instructions. Real-time quantitative PCR was conducted with AceQ qPCR SYBR Green Master Mix (Vazyme, Nanjing, China). All samples underwent triplicate testing, and average CT values were determined. qPCR primers were constructed using Premier 6.0 software referencing GenBank sequence data, with β-actin employed as the housekeeping gene (Table S1). All oligonucleotides were commercially synthesized by TsingKe (Beijing, China).

### 2.3. Western Blot Analysis

Post-NAT10 knockdown or PCV2 treatment, cells were gathered and rinsed with cold PBS (4 °C). Lysis was executed in buffer solution (Applygen, Beijing, China) containing 100× phosphatase and protease inhibitors (TargetMOI, Boston, MA, USA). After 20 min on-ice incubation, samples were spun at 12,000 rpm (15 min, 4 °C) and supernatants obtained. Protein levels were assessed by BCA assay (Yeason, Shanghai, China). Samples were mixed with 5× loading buffer (Vazyme, Nanjing, China), boiled at 98 °C (10 min), and frozen at −20 °C.

Protein samples were resolved on 10% SDS–PAGE gels and electroblotted onto polyvinylidene fluoride (PVDF) membranes (EMD Millipore, Billerica, MA, USA). The membrane was blocked with NcmBlot Blocking Buffer (NCM, Suzhou, China) for 10 min at room temperature, and incubated with the primary antibodies including: anti-mouse PCV2 Cap (GeneTex, San Antonio, TX, USA), anti-pig HSP90 (Proteintech, Wuhan, China), anti-mouse NAT10 (Proteintech, Wuhan, China), and anti-mouse NR1H4 (Abcam, Shanghai, China). Post-TBST washing, membranes were exposed to species-specific HRP-conjugated second antibodies (Proteintech, Wuhan, China) for 2 h under room temperature conditions. Primary and secondary antibodies were applied at final concentrations of 1 µg/mL and 0.02 µg/mL, respectively. Protein signals were detected via enhanced chemiluminescence (ECL) system (Tanon, Shanghai, China).

### 2.4. RNA Interference

Three small interfering RNA (siRNA) sequences targeting NAT10 (siNAT10-453, siNAT10-161, and siNAT10-982) and a negative control (siNC) (Table S2) were chemically synthesized by GenePharma (Suzhou, China). PK15 cells were plated into six-well plates at 5 × 10^5^ cells/well and cultivated for 24 h to achieve ~70% confluency. Transfection was performed using 10 μL Lipofectamine 2000 reagent (Invitrogen, Carlsbad, CA, USA) with either 5 μg plasmid DNA or 100 pmol siRNA in Opti-MEM medium according to standard procedures. At 6 h post-transfection, the medium was replaced with fresh complete medium. After 48 h incubation, cells were harvested for RNA and protein isolation, followed by qRT-PCR and Western blot analyses to evaluate NAT10 expression. PK15 cells exhibiting effective NAT10 silencing were then selected for further experiments.

### 2.5. Indirect Immunofluorescence Assay (IFA)

PCV2d-infected cells were harvested at multiple time intervals for immunofluorescence analysis. Following PBS washing, cells underwent fixation with 4% paraformaldehyde at 37 °C for 30 min. Membrane permeabilization was achieved using 0.5% Triton X-100 (Vazyme, Nanjing, China) for 15 min, after which samples were blocked with 5% BSA (bovine serum albumin; Solarbio, Beijing, China) at 37 °C for 2 h. Specimens were subsequently probed with PCV2 Cap antibody (PCV2 Cap; VMRD, Pullman, WA, USA), and then incubated with a fluorescent-labeled goat anti-mouse IgG secondary antibody (Huabio, Hangzhou, China). Nuclear counterstaining was performed using DAPI (4′,6-diamidino-2-phenylindole) for 5 min, and slides were mounted using antifade mounting medium (Biosharp, Beijing, China). Images were acquired via fluorescence microscopy (Leica Microsystems, Wetzlar, Germany).

### 2.6. RNA Sequencing and Data Analysis

Following 48 h PCV2 infection, PK15 cells from both control (siNC, *n* = 3) and NAT10-silenced groups (siNAT10, *n* = 3) were harvested. RNA isolation was performed using TRIzol reagent, with integrity assessment conducted via 1% formaldehyde-denaturing agarose gel electrophoresis. An ND-1000 spectrophotometer determined RNA quantity and purity. Qualified RNA samples underwent reverse transcription to generate double-stranded cDNA, followed by Illumina HiSeq 2500 sequencing (Oebiotech, Shanghai, China). Genes exhibiting differential expression (DEGs) were identified using a corrected *p*-value threshold of < 0.05. Gene Ontology (GO) enrichment utilized GOseq, whereas Kyoto Encyclopedia of Genes and Genomes (KEGG) pathway analysis employed KOBAS (version 3.0). Enrichment significance was determined at adjusted *p* < 0.05. Protein–protein interaction (PPI) networks for DEGs were generated and displayed through Cytoscape 3.10.4 [33].

### 2.7. Statistical Analysis

Data analysis was conducted using SPSS 18.0 (version 18.0, SPSS Inc., Chicago, IL, USA) and GraphPad Prism 9.0 (GraphPad Software, La Jolla, CA, USA). Transcript abundance was quantified via the 2^−ΔΔCt^ method. Results are presented as mean ± standard deviation (SD), with group comparisons performed through Student’s *t*-test. Statistical significance was defined as *p* < 0.05.

## 3. Results

### 3.1. Establishment of PCV2-Infected PK15 Cell Model

**Figure 1:**
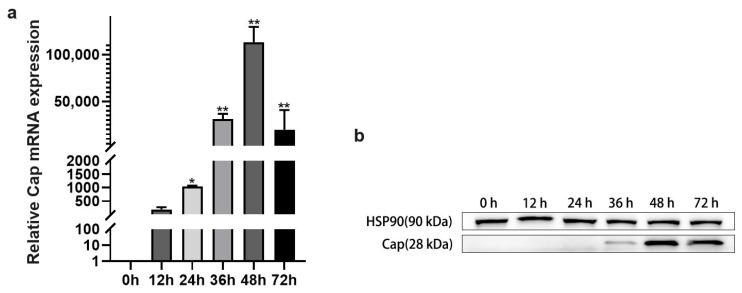
Construction of PCV2 infection model (MOI = 1) in PK15 cells. (**a**) Temporal profile of relative Cap mRNA expression in PK15 cells following PCV2 infection at indicated time intervals (0, 12, 24, 36, 48, and 72 h). (**b**) Cap protein abundance in PCV2-challenged PK15 cells across different time courses. Values represent mean ± SD from triplicate experiments, * *p* < 0.05, ** *p* < 0.01.

To investigate the replication and pathogenic mechanisms of PCV2 in host cells, a PK15 cell infection model was established with PCV2 at different time points (0 h, 12 h, 24 h, 36 h, 48 h, and 72 h). Following infection, the expression of the Cap gene was analyzed by qPCR and Western blot at each time point. As shown in Figure 1, Cap mRNA levels increased progressively from 0 to 48 h, reaching a peak at 48 h before declining at 72 h. Consistently, Western blot analysis revealed elevated Cap protein expression up to 48 h. These results confirm the successful establishment of a PCV2-infected PK15 cell model.

### 3.2. Down-Regulated NAT10 Expression in PK15 Cells After PCV2 Infection

**Figure 2:**
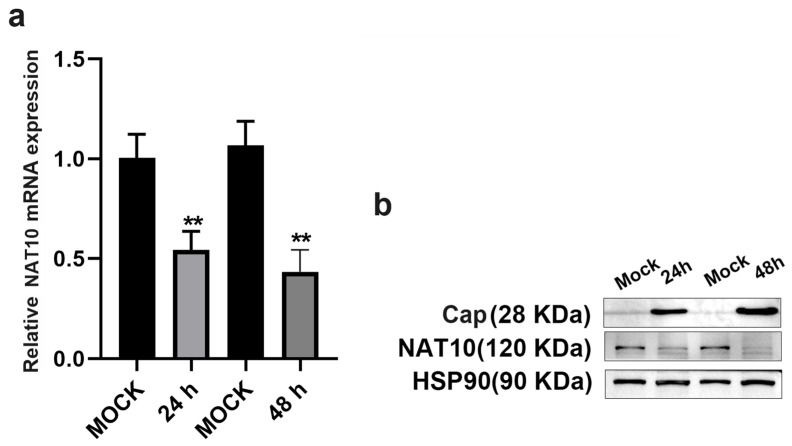
NAT10 expression in PK15 cells after PCV2 infection. (**a**) Relative NAT10 mRNA expression was analyzed by qRT-PCR at 24 h and 48 h post-treatment compared with the mock control (normal PK15 cells) group. (**b**) Western blot analysis of Cap and NAT10 protein levels at the indicated time points. HSP90 was used as an internal control. Values represent mean ± SD from triplicate experiments, ** *p* < 0.01.

To preliminarily assess the role of the ac^4^C modification marker in cellular resistance to PCV2 infection, the expression of the ac^4^C marker NAT10 was examined in PK15 cells following PCV2 infection. As shown in Figure 2a, a statistically significant decrease in NAT10 mRNA expression was observed at 24 h and 48 h post-infection compared to the control group (*p* < 0.01), as determined by qPCR assays, suggesting downregulation at the transcriptional level. Consistently, Western blot analysis demonstrated a time-dependent decrease in NAT10 protein expression, aligning with the qRT-PCR results. These findings suggest that NAT10 may serve as a key regulator influencing host susceptibility to PCV2 infection.

### 3.3. NAT10 Is Required for the PCV2 Replication Regulation in PK15 Cells

**Figure 3:**
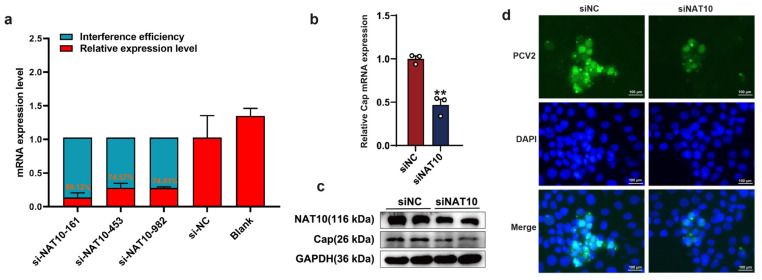
NAT10 is essential for regulating PCV2 replication in PK15 cells. (**a**) The silencing efficacy of NAT10 in PK15 cells was assessed via quantitative PCR analysis. (**b**) Relative Cap mRNA expresssion following NAT10 depletion (si-NAT10-161) was quantified by qPCR. (**c**) Cap protein levels after NAT10 knockdown (si-NAT10-161) were analyzed through Western blotting. Two lanes for siNC and siNAT10 represent two replicates. (**d**) Indirect immunofluorescence staining: DAPI (blue) labels cell nuclei; anti-PCV2 Cap antibody (green) marks viral Cap protein. Images were captured using fluorescence microscopy (magnification: 100×, scale bar = 100 µm). Values represent mean ± SD from triplicate experiments, ** *p* < 0.01.

To elucidate the mechanism underlying NAT10-mediated regulation of PCV2 replication, we investigated the effect of NAT10 expression on viral replication in PK15 cells. Three siRNA constructs targeting NAT10 were designed, among which siNAT10 exhibited the highest interference efficiency (89.12%), as verified by qPCR (Figure 3a). Following NAT10 knockdown, PCV2 replication was evaluated by qPCR, immunofluorescence assay (IFA), and Western blot analysis. qPCR results showed that NAT10 silencing significantly decreased the expression of the PCV2 Cap gene (Figure 3b). Western blot analysis further confirmed a marked reduction in PCV2 protein levels after NAT10 knockdown (Figure 3c). Consistently, IFA results revealed stronger PCV2 Cap protein fluorescence in the control group than in the siNAT10-treated cells (Figure 3d). Collectively, these findings Overall, these findings reveal that NAT10 functions as a proviral factor essential for PCV2 replication, whereas its loss significantly augments the capacity of PK15 cells to resist PCV2 infection.

### 3.4. Comparative Transcriptome Analysis of PK15 Cells with NAT10 Knockdown

**Figure 4:**
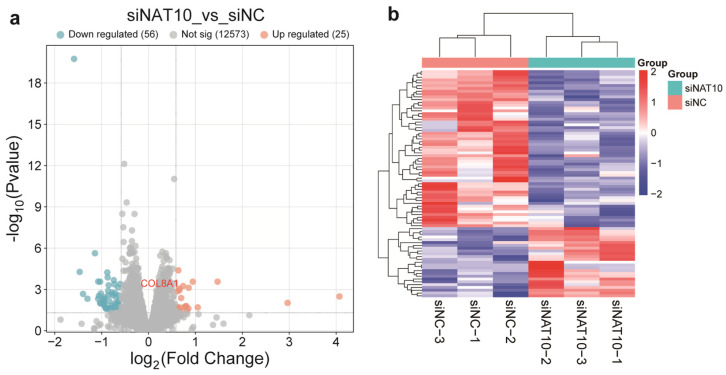
Transcriptomic profiling comparing siNAT10 and siNC samples. (**a**) Volcano diagram depicting the pattern of modulated genes. The horizontal axis displays log_2_ fold values, while the vertical axis shows −log_10_(*p*-value). Genes exhibiting increased expression appear in red, those with decreased expression in blue, and unchanged genes in gray. siNAT10 represents the PK15 cells with si-NAT10-161 vector. (**b**) Color-coded matrix presenting unsupervised clustering of DEGs across siNAT10 and siNC groups. Horizontal entries correspond to individual genes; vertical entries represent independent biological replicates. Red and blue hues indicate elevated and reduced transcript abundance, respectively.

To investigate the molecular mechanism by which NAT10 regulates PCV2 replication in PK15 cells, comparative transcriptome sequencing was performed on NAT10 knockdown (siNAT10, *n* = 3) and control (siNC, *n* = 3) groups. A total of 46.13, 40.65, and 46.88 million raw reads were obtained for siNAT10-1, siNAT10-2, and siNAT10-3, respectively, and 47.05, 43.10, and 48.47 million reads for siNC-1, siNC-2, and siNC-3. After quality filtering, approximately 96.93% (95.88–97.47%) of the clean reads were successfully mapped to the pig reference genome, with 91.29–93.34% mapping uniquely and 3.86–4.59% showing multiple alignments (Table S3). Differential expression analysis revealed 81 differentially expressed genes (DEGs) between the siNAT10 and siNC groups, among which 25 genes were significantly upregulated in the NAT10 knockdown group (Figure 4, Table S4). These DEGs may represent potential downstream targets involved in NAT10-mediated regulation of PCV2 replication.

**Figure 5:**
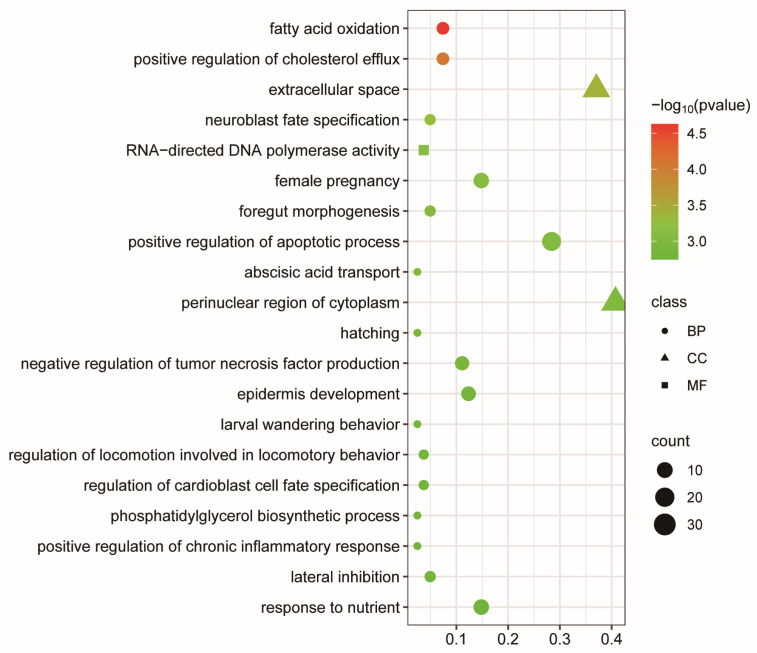
Gene ontology (GO) enrichment. Bubble plot showing significantly enriched GO terms in the categories of biological process (BP), cellular component (CC), and molecular function (MF). The y-axis lists the GO terms, while the x-axis shows the Gene Ratio (proportion of DEGs associated with each GO term). The color scale represents the −log_10_(*p*-value), indicating the statistical significance of enrichment, with red denoting higher significance.

**Figure 6:**
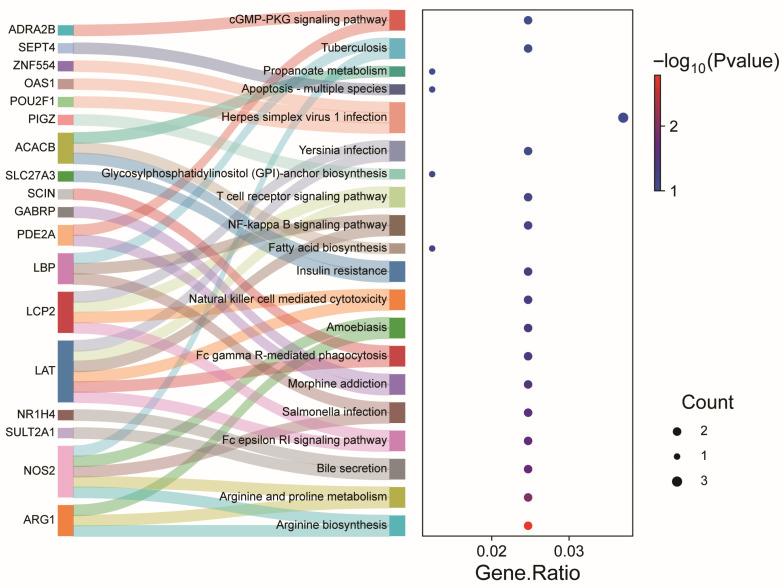
KEGG-based pathway enrichment profiling of modulated genes. Composite Sankey-bubble visualization displaying statistically significant KEGG pathways. The Sankey plot (**left**) links individual DEGs (**left**) to their associated KEGG pathways (**right**), with colored ribbons indicating the relationships between genes and pathways. The bubble plot (**right**) displays the enrichment results, where the x-axis represents the Gene Ratio, the bubble size indicates the number of DEGs involved in each pathway, and the color scale denotes statistical significance (−log_10_(*p*-value)).

To gain deeper insights into the functional implications of DEGs following NAT10 depletion, we performed Gene Ontology (GO) and Kyoto Encyclopedia of Genes and Genomes (KEGG) pathway enrichment analyses. As shown in Figure 5, the DEGs were categorized into 20 GO functional groups, including the perinuclear region of the cytoplasm, extracellular space, and positive regulation of apoptotic processes. KEGG enrichment analysis demonstrated that the majority of these differentially expressed genes were associated with immune-related signaling cascades, including T cell receptor signaling, NF-κB signaling, Fc epsilon RI signaling, and Fc gamma R-mediated phagocytosis pathways (Figure 6, Table S5). Collectively, these results suggest that NAT10 may modulate PCV2 replication through downstream immune and apoptotic regulatory pathways, as revealed by transcriptome analysis.

### 3.5. NR1H4 Probably Acts as a Potential Target of NAT10 in PCV2 Infection

**Figure 7:**
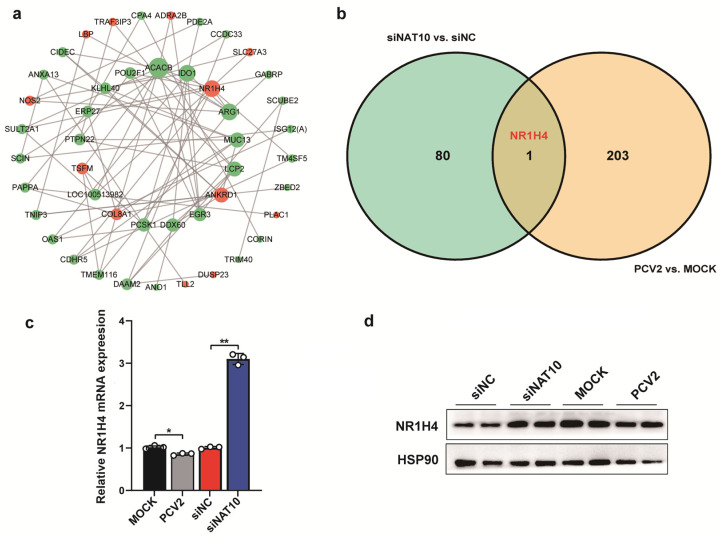
Potential targets identification of *NAT10* in PCV2 infection. (**a**) Protein–Protein Interaction (PPI) network generated from differentially modulated genes. Network nodes symbolize proteins, and connecting lines symbolize protein–protein interaction. Gene expression status is indicated by node color: green for decreased expression and red for increased expression. (**b**) Venn Diagram of differentially expressed genes in PCV2-infected kidney tissues and NAT10-knockdown PK15 cells. (**c**) qPCR results quantifying the relative NR1H4 mRNA expression in different treatment groups (PCV2 vs. MOCK, siNAT10 vs. siNC). (**d**) Western blot analysis of NR1H4 protein. Two lanes for each represent two replicates. Values represent mean ± SD from triplicate experiments, * *p* < 0.05, ** *p* < 0.01.

To further clarify the potential target of *NAT10* in PCV2-infected PK15 cells, we conducted the protein–protein interaction network of DEGs between siNAT10 group and siNC group. As shown in Figure 7a, some proteins including MUC13, NR1H4, COL8A, DDX60, etc., located at the core of the network. In our previous study, we performed the transcriptome of kidney tissues in 28-day-old piglets after PCV2d-P58 (10^7^ TCID_50_/mL × 10 mL) challenge, and obtained 204 DEGs related to PCV2 infection. On this basis, we screened out a common DEG, namely *NR1H4*, in PCV2-infected kidney tissues and *NAT10*-knockdown PK15 cells (Figure 7b). Further qRT-PCR validation (Figure 7c) showed that NR1H4 expression was significantly upregulated in NAT10-silenced cells (*p* < 0.01) but downregulated in PCV2-infected kidney tissues (*p* < 0.05). Consistent with these findings, Western blot analysis (Figure 7d) confirmed similar trends at the protein level. Collectively, these results suggest that NR1H4 is a downstream regulatory target of NAT10, as supported by integrated transcriptomic and proteomic analyses.

## 4. Discussion

Epitranscriptomic modifications have been widely investigated in various viral life cycles, including those of HIV, EV71, influenza virus, flaviviruses, coronaviruses, and herpesviruses. Among these, methylation modifications such as m^6^A, m^1^A, m^5^C, guanosine methylation, and 2′-O-methylation have been extensively characterized [34,35,36,37,38]. However, the functional significance of RNA acetylation during viral infection has been poorly characterized. Here, we established that NAT10 serves an essential function in modulating PCV2 infection. Previous studies have highlighted the involvement of other epigenetic regulators in PCV2 pathogenesis. For example, Shan et al. reported that the DNA methylation inhibitor 5-azacytidine activates the MAPK signaling pathway, thereby enhancing PCV2 replication and promoting inflammatory and apoptotic responses [29]. Du et al. showed that DNMT3B inhibits PCV2 replication by targeting TMEM37 to modulate Ca^2+^ influx in PK15 cells [30]. Similarly, Li et al. found that m^6^A modification facilitates PCV2 replication in PK15 cells [32]. Collectively, these findings underscore the importance of epigenetic modifications in PCV2 infection and replication. However, the role of RNA ac^4^C modification in PCV2 biology has not been previously defined. Our study revealed that NAT10 expression was significantly downregulated at 24 h and 48 h post-PCV2 infection, suggesting its close association with viral replication. Functional assays further confirmed that NAT10 silencing markedly suppressed PCV2 replication in PK15 cells. Together, these results indicate that NAT10 contributes to PCV2 infection and may serve as a potential molecular marker and regulatory factor during viral replication.

Recent investigations, including data from our laboratory, highlight NAT10 as a key determinant of virus–host interactions and innate immune modulation [9,10,11,39]. Yet, its specific mechanistic role in PCV2 pathogenesis remains uncharacterized. The current study demonstrates that NAT10 influences viral replication by engaging immune signaling circuits, notably the T cell receptor and NF-κB pathways. Previous studies have shown that PCV2 activates NF-κB signaling and promotes inflammatory responses through the circPDCD4/miR-21/PDCD4 axis in PK15 cells [40]. As an RNA acetyltransferase, NAT10 functions as the primary “writer” of N4-acetylcytidine (ac^4^C) modification, regulating the expression of target genes by influencing mRNA stability and translational efficiency [8,41]. Current research has revealed that NAT10-mediated ac^4^C modification contributes to the pathogenesis of various diseases, including cancers, by altering gene expression patterns through site-specific ac^4^C modifications [42,43,44]. For instance, NAT10 targets ULK1 in neutrophils, and its downregulation leads to ULK1 mRNA degradation and activation of the STING–IRF3 pathway, thereby enhancing NLRP3 inflammasome-mediated pyroptosis [14,45]. Although additional interferon-related genes, such as CAPN2 and ASS1, have been identified as NAT10-regulated targets [46,47], their precise functional mechanisms remain incompletely understood. In the present study, comparative transcriptome analysis was used to identify potential NAT10 target genes in PK15 cells. Bioinformatics and experimental validation revealed NR1H4 as a likely downstream effector of NAT10 during PCV2 infection. Previous research demonstrated that NR1H4 exerts neuroprotective effects in Parkinson’s disease by suppressing astrocyte activation and neuroinflammation via the CEBPβ/NF-κB pathway [48]. Moreover, NR1H4 has been implicated in anti-inflammatory responses in several disease models. Our data showed that NR1H4 expression was significantly downregulated in PCV2-infected kidney tissues, suggesting that NAT10 may regulate antiviral immunity through NR1H4-mediated signaling. In future studies, we aim to elucidate the detailed mechanism by which NAT10-mediated ac^4^C modification regulates NR1H4 expression and to systematically verify the role of NAT10 in host resistance to PCV2 infection.

## 5. Conclusions

In summary, this study is the first to elucidate the role and molecular mechanism of NAT10 in regulating PCV2 infection. Our findings demonstrate that NAT10 plays a crucial role during PCV2 infection of host cells and that its knockdown significantly suppresses viral replication in PK15 cells. Transcriptomic analysis further revealed that NAT10 regulates PCV2 replication through immune-related signaling pathways and identified NR1H4 as a potential downstream target. Collectively, these results provide new insights into the epitranscriptomic regulation of PCV2 infection and suggest that NAT10 could act as a valuable biomarker and intervention target in the context of PCV2-associated diseases.

## Data Availability

The original contributions presented in this study are included in the article/Supplementary Materials. Further inquiries can be directed to the corresponding author(s).

## Supplementary Materials

The following supporting information can be downloaded at https://www.mdpi.com/article/10.3390/vetsci12121160/s1, Table S1: Primer information for the RT-qPCR; Table S2: Sequence information of siRNA for gene; Table S3: Statistics for the sequencing data; Table S4: Differentially expressed genes between siNAT10 and siNC control groups (siNAT10 vs. siNC); Table S5: List of KEGG pathways obtained by enrichment analysis.

## Abbreviations

PCV2	porcine circovirus type 2
NAT10	N-acetyltransferase 10
qRT-PCR	quantitative real-time PCR
DEGs	differential expression genes
PK15	porcine kidney cells
Cap	capsid protein
GO	Gene Ontology
KEGG	Kyoto Encyclopedia of Genes and Genomes
SD	standard deviation
IFA	indirect fluorescent antibody
